# The Associations Between Physical Activity, Body Perception, and Self-Rated Health in Korean Adults: An Analysis of the 2023 Korean Community Health Survey

**DOI:** 10.3390/medicina61111898

**Published:** 2025-10-23

**Authors:** Geun-Kook Kim, Su-Yeon Roh, Sung-Ho Hwang

**Affiliations:** 1 Department of Sports Rehabilitation, Jaeneung University, Incheon 22573, Republic of Korea; 3k4412@jeiu.ac.kr; 2 Department of Exercise Rehabilitation, Gachon University, Incheon 21936, Republic of Korea; 3 Department of Integrated Alternative Medicine, Shinhan University, Uijeongbu 11644, Republic of Korea

**Keywords:** physical activity, body perception, subjective health, health behavior, Korean adults

## Abstract

*Background and Objectives*: This study examined associations between physical activity, body perception, and self-rated health (SRH) in a nationally representative sample of Korean adults. *Materials and Methods*: This study analyzed data from the 2023 Korea Community Health Survey (n = 228,249 adults aged ≥19). Variables included Body Mass Index (BMI), body perception, and participation in walking and stretching. Complex sample *t*-tests and one-way ANOVA were used to examine group differences in BMI, body perception, and self-rated health (SRH). Comparisons were made across gender, age groups, residential environment (metropolitan vs. non-metropolitan), and household size. *Results*: Mean BMI was highest in individuals in their 30s and was lowest in those aged 80 and above (*p* < 0.001). Females reported lower BMIs and more positive SRH than males (*p* < 0.001). Metropolitan residents and individuals in larger households showed higher physical activity rates and more favorable SRH (*p* < 0.001). SRH and physical activity declined with age, while exercise participation was higher among individuals with higher BMI or self-perceived overweight status (*p* < 0.001). Although the direction of this association should be interpreted with caution. *Conclusions*: Age, gender, residence, and household composition significantly influenced physical activity and SRH. These findings highlight the need to prioritize interventions for older adults, single-person households, and non-metropolitan populations.

## 1. Introduction

South Korea is facing rapid population aging due to persistently low birth rates and an increasing proportion of older adults. These demographic changes have led to substantial social and economic challenges, including rising medical costs and a shrinking working-age population [1]. Consequently, maintaining public health through individual health behaviors and subjective health assessments has become a critical task for sustainable social development [2,3]. Self-rated health (SRH), defined as an individual’s subjective evaluation of their own health, is a well-established predictor of actual health status and mortality risk [4]. Previous studies show that people who rate their subjective health status as “poor” have a significantly higher risk of death than those who rate it as “good,” and this finding has been consistently confirmed in long-term follow-up studies [5,6]. In this regard, SRH can be an important goal of health promotion policies and personalized health management strategies.

Health behaviors refer to activities and habits that individuals choose to maintain and promote their health, such as regular physical activity and proper eating habits [7]. Public health guidelines recommend at least 150 min of moderate physical activity per week for adult health [8,9]. Additionally, stretching plays an important role in preventing physical decline caused by aging because it improves joint mobility and muscle flexibility [10]. Furthermore, regular walking exercise is considered an important factor in improving SRH assessments. For example, a study of more than 60,000 Brazilian adults found that those who walked for more than 150 min per week were 28% more likely to report being in good health than those who did not, and those who walked for more than 300 min per week were 52% more likely to report being in good health [11]. In particular, walking ability and speed are closely related to SRH, especially among older adults, and older adults who have difficulty walking are significantly more likely to evaluate their health as poor [12].

Moreover, socioeconomic status and lifestyle factors play crucial roles in shaping both physical activity levels and overall health perception. Individuals with higher income and educational attainment tend to participate more frequently in leisure-time physical activities, while those with lower socioeconomic status often experience environmental and time constraints that limit exercise opportunities [13]. Such disparities may partially explain health inequalities observed across social groups. In addition, regular physical activity has been shown to significantly extend life expectancy by improving cardiovascular health, metabolic function, and mental well-being [14]. Furthermore, eating habits also influence perceived and actual health, particularly among older adults, whose nutritional intake often declines due to physiological and economic factors [15]. These social and behavioral factors together interact with physical activity to affect subjective health perception and overall longevity.

The relationship between physical activity and SRH has been well established in large-scale studies [16]. Adults who engage in regular physical activity are significantly more likely to rate their health positively than those who do not [17], and physical function levels are closely associated with SRH, particularly among older adults [4,18]. In contrast, objective body mass index (BMI) and subjective body perception represent two distinct aspects of body weight. BMI is an objective anthropometric indicator calculated from height and weight, whereas body perception refers to an individual’s self-perceived body weight status (e.g., “too fat” or “too thin”), reflecting psychological and cultural influences rather than biological measures. For example, a study of Korean older adults found that those who perceived their bodies as “too fat” or “too thin” were more likely to report poorer SRH, even within the same BMI category [19]. These findings indicate that physical activity and body perception each influence how individuals evaluate their health, beyond what is captured by objective indicators such as BMI.

We hypothesized that higher physical activity and more positive body perception would be associated with better SRH, after accounting for demographic factors. Therefore, this study aims to clarify the relationship between physical activity, body perception, and SRH among Korean adults, while considering key demographic characteristics such as age, gender, residential area, and household type. In doing so, we sought to provide empirical evidence to inform the development of tailored health-promotion strategies in Korean society.

## 2. Materials and Methods

### 2.1. Data Source and Study Subjects

**Figure 1:**
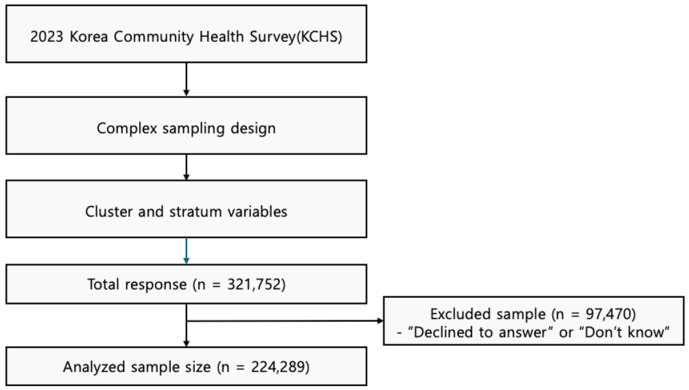
Study flow.

This study used data collected from the 2023 Korean Community Health Survey (KCHS), which was conducted by the Korea Disease Control and Prevention Agency (KDCPA). The 2023 KCHS is a nationally representative sample survey conducted at health centers in 17 regions (Statistics Korea approval number 117075), targeting approximately 900 adults aged 20 and older as of 2023. The survey is divided into individual surveys and household surveys. Household surveys are conducted with one representative member of each household, while individual surveys are conducted separately with all members of each household. The CAPI process involved home visits by an interviewer, internet-based responses from the subject, and, if assistance was needed during the survey, the interviewer provided additional explanations to ensure clear responses. The 2023 KCHS period was from 16 May 2023 to 31 July 2023, and the survey method used was Computer Assisted Personal Interviewing (CAPI). The KCHS was conducted in Korean, targeting South Korean adults aged 20 years and older. All participants were residents of South Korea. Typical survey items for household surveys include location and household, while typical survey items for individual surveys include sex, age, physical activity, SRH [20]. We focus on physical activity participation, BMI, body perception, and SRH according to gender, age, and living arrangements among adults. KCHS data are important as representative data for the entire population of South Korea, which means that the analysis results are statistically reliable. Since 2016, KCHS’s Institutional Review Board (IRB) has been excluded from review because it does not qualify as human subject research, based on the IRB number (2016-10-01-P-A) approved by the Korea Disease Control and Prevention Agency. The overall research design and analysis process of this study are summarized in Figure 1.

### 2.2. Study Participants

The study subjects are presented in Table 1. A total of 321,752 South Korean adults responded to the survey. To ensure data completeness and reliability, we included adults aged 20 years and older who completed all relevant items on physical activity, body perception, and SRH. Respondents with missing data in any of these key variables or implausible anthropometric values were excluded from the analysis. After applying these criteria, final analytic sample was 228,249. Of these, 104,995 (46.0%) were male, and 123,254 (54.0%) were female. By age group, 21,446 individuals (9.4%) were aged 20–29, 24,240 (10.6%) were aged 30–39, 33,659 (14.8%) were aged 40–49, 42,516 (18.6%) were aged 50–59, 51,449 (22.5%) were aged 60–69, 34,756 (15.2%) were aged 70–79, and 20,183 (8.8%) were aged 80 or older. Regarding residence, 75,016 individuals (32.9%) lived in metropolitan areas, while 153,233 (67.1%) lived in non-metropolitan areas. For household size, 40,471 (17.7%) were single-person households, 79,222 (34.7%) were two- to three-person households, 96,734 (42.4%) were four- to six-person households, and 11,822 (5.2%) were households with seven or more members. The number of people participating in leisure activities was 71,824 (31.5%), while 156,425 (68.5%) did not participate. The number of people participating in walking was 189,430 (83.0%), while 38,819 (17.0%) did not participate. Among those who participated in stretching exercises, 127,616 (55.9%) participated, while 100,633 (44.1%) did not.

### 2.3. Measure Variables

The 2023 KCHS included 17 areas and a total of 145 survey questions. However, to achieve the purpose of this study, we used variables such as participation and frequency of walking and stretching exercises in the physical activity area, in addition to general characteristics of the research subjects. Additionally, in the obesity and weight control domain, we calculated BMI based on self-reported height and weight, and used the body perception variable. Because BMI was calculated from self-reported data, it may be subject to reporting bias due to potential discrepancies between reported and actual values. Finally, in the activity limitation and quality of life domain, we used SRH level as a variable.

#### 2.3.1. Physical Activity

Physical activity types were classified as walking and stretching.

(i) To assess the level of participation in walking, we asked the following question: Based on walking for transportation and exercise, including commuting to work and school, “How many days in the past week did you walk for at least 10 min at a time?” Respondents selected a number from zero to seven. Walking for at least 10 min at a time has been widely used as a criterion for physical activity in population-based surveys, including the Korea Community Health Survey and national health guidelines, which recognize that even short bouts of walking contribute to overall health benefits [21].

(ii) To assess the level of participation in stretching, we asked: “How many days in the past week did you do flexibility exercises such as stretching and calisthenics?” Respondents chose from the following options: “Not at all,” “1 day,” “2 days,” “3 days,” “4 days,” and “5 days or more.” Flexibility exercises include static and dynamic stretches, joint mobility movements, and light bodyweight calisthenics (e.g., arm/leg stretches, torso twists) intended to improve movement range and muscle elongation [22].

For the present analysis, both walking and stretching variables were treated as categorical variables (Yes/No), where “Yes” indicated participation in the activity at least one day per week and “No” indicated no participation. This categorization was applied to maintain consistency with other dichotomous indicators of physical activity in the KCHS dataset.

#### 2.3.2. BMI

The BMI calculation method divides weight by the square of height (kg/cm^2^). Therefore, to calculate BMI, weight (kg) and height (cm) are required. The question asked is, “What is your current height and weight?” and research subjects respond with the height and weight they currently know. Therefore, the BMI values calculated in this study do not represent the subjects’ actual measurements, but rather their self-reported BMI.

#### 2.3.3. Body Perception

The question used to assess body perception is “How would you describe your current body type?” Respondents are asked to choose from the following options: “(1) = Very thin,” “(2) = Slightly thin,” “(3) = Average,” “(4) = Slightly overweight,” and “(5) = Very overweight.” Therefore, a lower score indicates that the individual perceives their body type as thin, while a higher score indicates that the individual perceives their body type as overweight. This variable was treated as an ordinal variable.

#### 2.3.4. Self-Rated Health (SRH)

The content of the questions used to assess SRH is as follows: “How would you rate your health on a typical day?” The response options are “(1) = Very good,” “(2) = Good,” “(3) = Average,” “(4) = Poor,” and “(5) = Very poor.” Therefore, a lower number indicates that the respondent evaluates their SRH more positively.

### 2.4. Statistical Analysis

All statistical analyses were performed using SPSS version 28.0 (IBM Corp., Armonk, NY, USA). The KCHS sample was a sample survey rather than a census. Therefore, to reduce bias in the research results, we analyzed the data using a complex sampling design, which included cluster variables, stratum variables, and post-stratification weights [23]. All analyses were performed using the Complex Samples Module in SPSS 28.0, which accounts for stratification, clustering, and sampling weights. We performed descriptive statistics and frequency analysis to identify the general characteristics of the study participants. In addition, we used *t*-tests to analyze BMI, body perception, and SRH according to gender (male/female), residence area (metropolitan/non-metropolitan), and participation in physical activity (yes/no), as well as to examine differences in the frequency of participation in physical activity by gender and residence area. We used one-way ANOVA across age groups to examine differences in BMI, body perception, and SRH according to age (20–80s), household size (person), and frequency of physical activity participation. When we found significant differences in the ANOVA, we used Scheffé’s post hoc test to confirm differences between groups. Scheffé’s method was selected because it is a conservative test that effectively controls the family-wise Type I error rate in multiple comparisons, even with unequal sample sizes among groups [24]. We set all statistically significant differences at the 0.05 level (*p* < 0.05). All *p*-values were based on two-tailed tests.

## 3. Results

Table 2 shows the levels of BMI, body perception, and SRH according to the general characteristics of the study subjects, including sex, age, location, household, and physical activity participation.

The difference in BMI levels was greater in males than in females (*p* < 0.001). Regarding age, individuals in their 30s had the highest BMI, whereas those aged 80 and above had the lowest (*p* < 0.001). There was no significant difference in BMI based on location (*p* = 0.913). However, BMI was higher in households where people lived together compared to those where people lived alone (*p* < 0.001). Additionally, BMI was higher in people who did not participate in physical activity (*p* < 0.001). The 95% confidence intervals were 24.42–24.46 kg/m^2^ for men and 23.02–23.06 kg/m^2^ for women, indicating a high level of precision due to the large national sample size.

Body perception data show that males perceive themselves as thinner than females (*p* < 0.001). Regarding age, individuals in their 80s and older perceive themselves as the thinnest, whereas those aged 20–59 evaluate themselves as having the fattest body type (*p* < 0.001). For location, people living in metropolitan areas perceive themselves as having a heavier body type (*p* < 0.001). Considering household composition, people living alone evaluate themselves as having a thinner body type, while those in households with four to six people perceive themselves as having a heavier body type (*p* < 0.001). Regarding physical activity type, individuals who participate in leisure activities perceive themselves as more overweight, whereas those who report participating in walking and stretching activities evaluate their body type as leaner (*p* < 0.001).

SRH was higher among females than males (*p* < 0.001). Additionally, younger individuals tended to evaluate their health more positively (*p* < 0.001). Regarding location, people living in metropolitan areas perceived themselves as healthier (*p* < 0.001). For household size, households with 4–6 members evaluated their health most positively, whereas people living alone perceived their health more negatively (*p* < 0.001). Regarding physical activity type, those who participated in leisure activities evaluated their health positively. However, even individuals who did not participate in walking or stretching activities also reported positive perceptions of their health (*p* < 0.001).

In Table 2, many group differences were statistically significant; however, most two-group effects were negligible (r^2^ < 0.01). Residential area showed a small effect on walking (r^2^ = 0.017), and leisure-activity participation showed small effects on SRH (r^2^ = 0.015) and stretching frequency (r^2^ = 0.041; Table 3). Across multi-group factors, age had a large effect on SRH (η^2^ = 0.157) and a small-to-moderate effect on body perception (η^2^ = 0.054), while household size had a small-to-moderate effect on SRH (η^2^ = 0.031).

Table 3 presents the results of analyzing differences in physical activity participation levels (days per week) according to the general characteristics of the study subjects. For the first type of physical activity, walking, and general characteristics, we find that males participate more than females (*p* < 0.001). The frequency of walking participation by age group tends to decrease with increasing age (*p* < 0.001). Regarding location, metropolitan areas show a higher frequency of walking participation than non-metropolitan areas (*p* < 0.001). Households with four to six members participate more than other types of households (*p* < 0.001). For leisure activity, people who report participating in walking do so more frequently (*p* < 0.001).

When examining the participation frequency and general characteristics of stretching, the second type of physical activity, we find that females participate more frequently than males (*p* < 0.001). By age group, individuals in their 50s and 60s report the highest participation frequency, followed by those in their 40s, while those aged 80 and above report the lowest participation frequency (*p* < 0.001). Residents of metropolitan areas report higher participation frequency (*p* < 0.001). Households with 2–3 or 4–6 members report higher participation frequency than single-person households or households with seven or more members (*p* < 0.001). Individuals who report participating in leisure activities also report higher participation frequency in stretching (*p* < 0.001).

Walking frequency showed small effects of age and residence (η^2^ = 0.0099; r^2^ = 0.017), and participation in leisure activities showed a small effect (r^2^ = 0.011). Stretching frequency showed a small to medium effect of age (η^2^ = 0.017), and participation in leisure activities showed a relatively large effect (r^2^ = 0.040). Although the remaining factors were statistically significant, their effect sizes were very small (r^2^ or η^2^ < 0.002).

Table 4 shows the differences in BMI, body perception, and SRH according to the level of physical activity participation. The BMI level by frequency of walking participation is highest among those who participate 5–7 days per week, followed by those who participate 1–2 days per week and 3–4 days per week, while those who do not participate have the lowest BMI (*p* < 0.001). Individuals are most likely to be evaluated as thin when not participating in walking, followed by those who walk 5–7 days per week, whereas those who participate 1–2 days per week and 3–4 days per week are most likely to perceive their body shape as fat (*p* < 0.001). SRH is highest among those who walk 5–7 days per week, followed by those who walk 1–2 days per week and 3–4 days per week. Those who report not walking at all rate their health as the poorest (*p* < 0.001).

Both walking and stretching frequency showed significant differences in SRH (*p* < 0.001), with small effect sizes (η^2^ = 0.021–0.024). Differences in BMI and body perception across frequency categories were statistically significant, but small in magnitude (η^2^ < 0.004).

## 4. Discussion

This study analyzed differences in body mass index (BMI), body perception, SRH, and physical activity participation rates according to gender, age, and type of residence using data from the 2023 Korea Community Health Survey (KCHS). Through this analysis, we aimed to identify the effects of demographic characteristics and living environments in Korean society on health behaviors and perceptions, and to propose directions for future health promotion policies.

The analysis showed that BMI was highest among people in their 30s and lowest among those aged 80 and above. We interpret this as resulting from changes in eating habits and meal size, which are associated with decreased muscle mass, reduced bone density, and a decline in digestive function caused by aging [25,26]. BMI did not show significant differences between metropolitan and non-metropolitan residents; however, metropolitan residents tended to perceive themselves as having a fatter body type. Although males exhibited higher BMI values than females, they were more likely to perceive themselves as thinner. This discrepancy may be attributed to gender differences in body perception, where males tend to underestimate their body weight or perceive a larger body size as more acceptable or even desirable, while females often show greater body dissatisfaction and stricter standards of thinness [27]. We also observed that members of four- to six-person households tended to evaluate themselves as fatter than those in single-person households. This pattern may be partly explained by the greater social exposure experienced by people living in metropolitan areas, where individuals might be more aware of others’ evaluations and more likely to engage in social comparison, including with family members. Such social and environmental factors could influence how individuals perceive their own bodies [28,29]. In addition, the BMI of multi-person households was higher than that of single-person households, which may be because living with family or roommates leads to more regular meal times and increased energy intake from shared meals [30]. Eating together can encourage regular eating habits; however, it can also increase the likelihood of overeating, which may lead to an increase in BMI [31].

Females had lower BMIs than males and rated their SRH status more positively. In addition, SRH evaluations were more positive among younger age groups. This finding can be interpreted as older age groups having lower rates of social participation and higher rates of chronic pain and disease incidence, which lead to more negative SRH evaluations [32]. Metropolitan residents rated their SRH status more positively than non-metropolitan residents and participated in walking exercise at a higher rate. This result may be because metropolitan areas are well equipped with walking-friendly environments, such as parks and walking trails, and offer greater access to healthy diets and exercise programs [2]. Furthermore, metropolitan areas have high population densities, which makes it easier to form exercise groups, and there are fewer psychological barriers to participating in group activities [33,34].

Household type also affects health awareness and physical activity participation rates. Individuals living in multi-person households reported better SRH and higher participation in physical activity, including walking, compared with those living alone [35]. This effect may occur because a greater number of family members provides more social support, such as emotional support, information sharing, and physical assistance [36]. This environment encourages healthy behavior, which leads to higher SRH awareness [37,38]. In addition, in multi-person households, healthy lifestyles can be reinforced through mutual monitoring and encouragement of lifestyle habits among family members [39]. However, households with seven or more members may present limitations in analyzing overall trends because of the high proportion of older adults or infants in these families.

Participation in walking exercise decreased with age, which we interpret as being due to physiological factors, such as a decline in basic physical strength because of aging and an increased risk of degenerative joint disease [40]. In contrast, metropolitan residents participated in walking more than non-metropolitan residents. This can be interpreted as being due to metropolitan infrastructure designed to encourage participation in exercise, as well as greater environmental accessibility and more opportunities for social participation [41]. We found a significant difference in participation rates for stretching by age group. In particular, the highest percentage occurred among those in their 40s and 50s. This finding relates to the tendency of middle-aged people to prefer exercises that can be performed indoors, while younger people prefer active outdoor exercises, which explains the low participation rate for stretching [42].

In addition, the more frequently participants engaged in physical activity, the more positively they evaluated their SRH. This pattern is also observed in previous studies [43]. Higher BMI was also associated with more frequent participation in exercise. This relationship may reflect self-motivated efforts to manage body weight or medical recommendations to increase physical activity for weight control and metabolic health. Participants who perceived themselves as overweight were also more likely to engage in exercise, suggesting that awareness of being overweight may encourage physical activity as a preventive behavior [44]. Furthermore, recognizing exercise as a healthy behavior leads to more positive assessments of SRH as exercise frequency increases. However, because this study used self-reported BMI rather than physical measurements, the reported figures may not be accurate. In addition, because BMI is calculated only from weight and height, it is difficult to conclude that a high BMI necessarily leads to negative outcomes.

Older adults aged 80 and above reported the lowest SRH and generally participated less in walking, stretching, and leisure activities. This finding suggests that aging is associated with a decline in health and physical activity, which can lead to a lower quality of life [45]. The decline in health among older adults increases healthcare demands and caregiving burdens within families, emphasizing the need for public health strategies that promote active and healthy aging [46]. Therefore, as South Korea becomes an aging society, policy interventions are needed to increase healthy life expectancy and promote active lifestyles among older adults. In particular, community-based physical activity programs, social participation initiatives, and health education tailored to different age and household groups may help extend healthy life beyond survival.

This study has several limitations. First, BMI values were self-reported based on participants’ own height and weight measurements, which may introduce reporting bias. Second, given the cross-sectional design, the possibility of reverse causality cannot be ruled out. While lower physical activity levels were associated with lower self-rated health, it is also possible that individuals in poorer health engaged in less physical activity. Third, multivariate regression analyses were not performed to adjust for potential confounding variables. Therefore, the results should be interpreted as unadjusted associations rather than causal relationships. Future studies employing regression-based models are needed to validate these associations while controlling relevant covariates.

## 5. Conclusions

In this large national survey, age, sex, residence, and household type were significantly associated with physical activity, body perception, and SRH. Findings highlight the need for targeted interventions for older adults, single-person households, and non-metropolitan residents. These findings highlight the importance of developing tailored health promotion programs that consider demographic and household characteristics, while improving environmental accessibility to reduce disparities between metropolitan and non-metropolitan areas. Community-based physical activity initiatives and family-centered health management strategies may contribute to promoting active aging and reducing health inequalities in Korean society.

## Figures and Tables

**Table 1:** General characteristics.

Variables			N (%)
Sex		Male	104,995 (46.0%)
		Female	123,254 (54.0%)
Age (years)		20–29	21,446 (9.5%)
		30–39	24,240 (10.6%)
		40–49	33,659 (14.8%)
		50–59	42,516 (18.6%)
		60–69	51,449 (22.5%)
		70–79	34,756 (15.2%)
		80 over	20,183 (8.8%)
Location		Metropolitan	75,016 (32.9%)
		Non-metropolitan	153,233 (67.1%)
Household (person)		Single	40,471 (17.7%)
		2–3	79,222 (34.7%)
		4–6	96,734 (42.4%)
		7 over	11,822 (5.2%)
Physical Activity	Leisure	Yes	71,824 (31.5%)
		No	156,425 (68.5%)
	Walking	Yes	38,819 (17.0%)
		No	189,430 (83.0%)
	Stretching	Yes	100,633 (44.1%)
		No	127,616 (55.9%)
Total			228,249 (100.0%)

**Table 2:** BMI, body perception and self-rated health according to general characteristics.

Variables			BMI (kg/m^2^)	Body Perception	Self-Rated Health
			M ± SD	M ± SD	M ± SD
Sex		Male	24.44 ± 3.27	3.20 ± 0.88	3.08 ± 0.93
		Female	23.04 ± 3.36	3.31 ± 0.87	3.06 ± 0.89
		*t*	100.500 ***	−30.721 ***	4.804 ***
		*r* ^2^	0.042	0.004	0.000
Age (years)		20–29 (a)	22.27 ± 4.15	3.28 ± 0.91	2.21 ± 0.78
		30–39 (b)	24.07 ± 4.10	3.47 ± 0.89	2.40 ± 0.75
		40–49 (c)	23.95 ± 3.61	3.45 ± 0.84	2.58 ± 0.72
		50–59 (d)	23.82 ± 3.12	3.35 ± 0.81	2.69 ± 0.77
		60–69 (e)	23.87 ± 2.91	3.27 ± 0.82	2.86 ± 0.85
		70–79 (f)	23.65 ± 3.02	3.08 ± 0.85	3.17 ± 0.92
		80 over (g)	22.53 ± 3.19	2.73 ± 0.92	3.51 ± 0.94
		*F*	573.432 ***	2168.954 ***	7071.262 ***
		Post hoc	g < a < f < d,e < c < b	g < f < a,e < b,c,d	a < b < c < d < e < f < g
		η^2^	0.015	0.054	0.157
GivemnLocation		Metropolitan	23.69 ± 3.48	3.31 ± 0.86	2.97 ± 0.87
		Non-metropolitan	23.68 ± 3.35	3.23 ± 0.88	3.12 ± 0.92
		*t*	0.110	18.414 ***	−36.591 ***
		*r* ^2^	0.000	0.001	0.006
Household (person)		Single (a)	23.53 ± 3.47	3.15 ± 0.91	3.01 ± 0.98
		2–3 (b)	23.70 ± 3.10	3.21 ± 0.85	2.88 ± 0.89
		4–6 (c)	23.72 ± 3.56	3.34 ± 0.87	2.62 ± 0.83
		7 over (d)	23.78 ± 3.58	3.28 ± 0.87	2.71 ± 0.87
		*F*	35.700 ***	537.727 ***	2397.381 ***
		Post hoc	a < b,c,d	a < b < d < c	c < d < b < a
		η^2^	0.000	0.007	0.031
Physical Activity	Leisure	Yes	23.81 ± 3.24	3.33 ± 0.81	2.86 ± 0.86
		No	23.63 ± 3.46	3.23 ± 0.90	3.17 ± 0.91
		*t*	12.383 ***	26.209 ***	−77.996 ***
		*r* ^2^	0.001	0.003	0.015
	Walking	Yes	23.72 ± 3.55	3.17 ± 0.95	3.05 ± 1.00
		No	23.68 ± 3.36	3.28 ± 0.86	2.73 ± 0.86
		*t*	2.382 *	−20.340 ***	59.482 ***
		*r* ^2^	0.000	0.002	0.015
	Stretching	Yes	23.77 ± 3.53	3.22 ± 0.93	2.94 ± 0.92
		No	23.62 ± 3.28	3.29 ± 0.83	2.66 ± 0.85
		*t*	10.212 ***	−16.805 ***	73.972 ***
		*r* ^2^	0.000	0.001	0.023

Note: * *p* < 0.05; *** *p* < 0.001. Data are expressed as M ± SD (mean ± standard deviation). a < b means group a lower than group b.

**Table 3:** Differences in physical activity participation levels according to general characteristics.

Variables		Walking (Days/Week)	Stretching (Days/Week)
		M ± SD	M ± SD
Sex	Male	4.27 ± 2.68	3.07 ± 2.19
	Female	4.20 ± 2.57	3.15 ± 2.11
	*t*	6.424 ***	−8.847 ***
	*r* ^2^	0.000	0.000
Age (years)	20–29 (a)	4.72 ± 2.36	3.06 ± 2.05
	30–39 (b)	4.39 ± 2.48	3.05 ± 2.03
	40–49 (c)	4.17 ± 2.52	3.11 ± 2.06
	50–59 (d)	4.18 ± 2.57	3.37 ± 2.14
	60–69 (e)	4.29 ± 2.64	3.32 ± 2.22
	70–79 (f)	4.27 ± 2.72	3.00 ± 2.22
	80 over (g)	3.56 ± 2.94	2.33 ± 2.02
	*F*	378.718 ***	669.146 ***
	Post hoc	g < c,d < e,f < b < a	g < a,b,f < c < d,e
	η^2^	0.009	0.017
Location	Metropolitan	4.70 ± 2.38	3.24 ± 2.11
	Non-metropolitan	4.01 ± 2.70	3.05 ± 2.16
	*t*	62.734 ***	19.674 ***
	*r* ^2^	0.017	0.001
Household (person)	Single (a)	4.19 ± 2.68	3.03 ± 2.17
	2–3 (b)	4.21 ± 2.69	3.15 ± 2.19
	4–6 (c)	4.28 ± 2.54	3.12 ± 2.10
	7 over (d)	4.20 ± 2.62	3.02 ± 2.13
	*F*	17.299 ***	32.896 ***
	Post hoc	a,b,d < c	a,d < b,c
	η^2^	0.000	0.000
Leisure activity	Yes	4.63 ± 2.42	3.75 ± 2.06
	No	4.06 ± 2.69	2.82 ± 2.12
	*t*	50.794 ***	98.790 ***
	*r* ^2^	0.011	0.040

Note: *** *p* < 0.001.

**Table 4:** Differences in BMI, body perception, and self-rated health according to frequency of participation in walking and stretching.

Variables		BMI (kg/m^2^)	Body Perception	Self-Rated Health
Physical activity	Frequency (days/week)	M ± SD	M ± SD	M ± SD
Walking	0 (a)	23.62 ± 3.43	3.17 ± 0.95	3.05 ± 1.00
	1–2 (b)	23.73 ± 3.39	3.33 ± 0.89	2.78 ± 0.84
	3–4 (c)	23.75 ± 3.30	3.31 ± 0.86	3.80 ± 0.85
	5–7 (d)	23.87 ± 3.28	3.25 ± 0.85	2.70 ± 0.87
	*F*	53.767 ***	233.635 ***	1600.172 ***
	Post hoc	a < b,c < d	a < d < b,c	d < b, c < a
	η^2^	0.000	0.003	0.021
Stretching	0 (a)	23.77 ± 3.53	3.22 ± 0.93	2.94 ± 0.92
	1–2 (b)	23.60 ± 3.43	3.34 ± 0.86	2.69 ± 0.82
	3–4 (c)	23.58 ± 3.27	3.30 ± 0.83	2.64 ± 0.82
	5–7 (d)	23.65 ± 3.22	3.25 ± 0.82	2.66 ± 0.88
	*F*	39.548	162.301 ***	1877.435 ***
	Post hoc	c,b < d < a	a < d < c < b	c < d < b < a
	η^2^	0.000	0.002	0.024

Note: *** *p* < 0.001.

## Data Availability

No new data were created or analyzed in this study.
